# An effective fluoroquinolone-based fluorescent probe for ultrasensitive Zn^2+^ detection exhibiting “turn-on” fluorescence

**DOI:** 10.1039/d6ra00838k

**Published:** 2026-10-13

**Authors:** Daniel Arulraj Abraham, Ravikumar Ayyanu, Ellairaja Sundaram, Balasubramani Govindasamy, A. Anand Babu Christus

**Affiliations:** a Green Technology, Department of Chemistry, SIMATS Engineering, Saveetha Institute of Medical and Technical Sciences, Saveetha University 602105 Tamil Nadu India aadaniel112@gmail.com danielarulraja.sse@saveetha.com; b Faculty of Science, Department of Chemistry, Chulalongkorn University Bangkok 10330 Thailand; c Department of Medicinal Chemistry, National Institute of Pharmaceutical Education and Research (NIPER) Guwahati & Department of Pharmaceuticals, Ministry of Chemicals and Fertilizers, Govt. of India Sila Katamur (Halugurisuk) Changsari, Kamrup Assam 781101 India; d Interdisciplinary Institute of Indian System of Medicine (IIISM), Directorate of Research, SRM Institute of Science and Technology Kattankulathur 603203 Tamil Nadu India; e Department of Chemistry, SRM Institute of Science and Technology, Ramapuram Campus India

## Abstract

Contamination of water and soil by heavy metal ions is a major global concern because these ions are toxic and persist in the environment for long periods. A new, effective fluorescent sensor based on a fluoroquinolone was developed for detecting zinc ions (Zn^2+^) with a turn-on signal. When Zn^2+^ ions are gradually added to the fluoroquinolone solution, the fluorescence intensity increases due to an internal charge transfer mechanism. We evaluated the detection of Zn^2+^ ions using the fluorescence probe in various solvents and across different pH conditions. The key results showed that the fluorescent probe demonstrated a strong preference for detecting Zn^2+^ ions in ethanol, maintaining accuracy despite the presence of other metal ions, and exhibited enhanced sensitivity. The developed sensor achieved a remarkably low detection limit of 1 nM for Zn^2+^ ions, outperforming earlier reported sensors. Fluorescence measurements exhibited a consistent linear correlation for Zn^2+^ concentrations between 1 nM and 15 µM. Furthermore, spectrophotometric data showed increased absorbance peaks upon addition of Zn^2+^ to the fluoroquinolone solution, confirming the creation of a fluoroquinolone–Zn^2+^ complex. The suggested probe was successfully applied to detect Zn^2+^ ions in wastewater and drinking water samples, achieving recovery rates ranging from 97.0% to 102.75%. More interestingly, binding to Zn^2+^ leads to significant changes in the fluorescence lifetime of the proposed probe, which can be used to visualize Zn^2+^ ions in A549 cells *via* Confocal fluorescence microscopy. The reported chemosensor with fluoroquinolone is an effective and economical system for real-time monitoring of Zn^2+^ ions.

## Abstract 2

Graphical representation of the zinc ion sensor by the probe.

## Graphical abstract

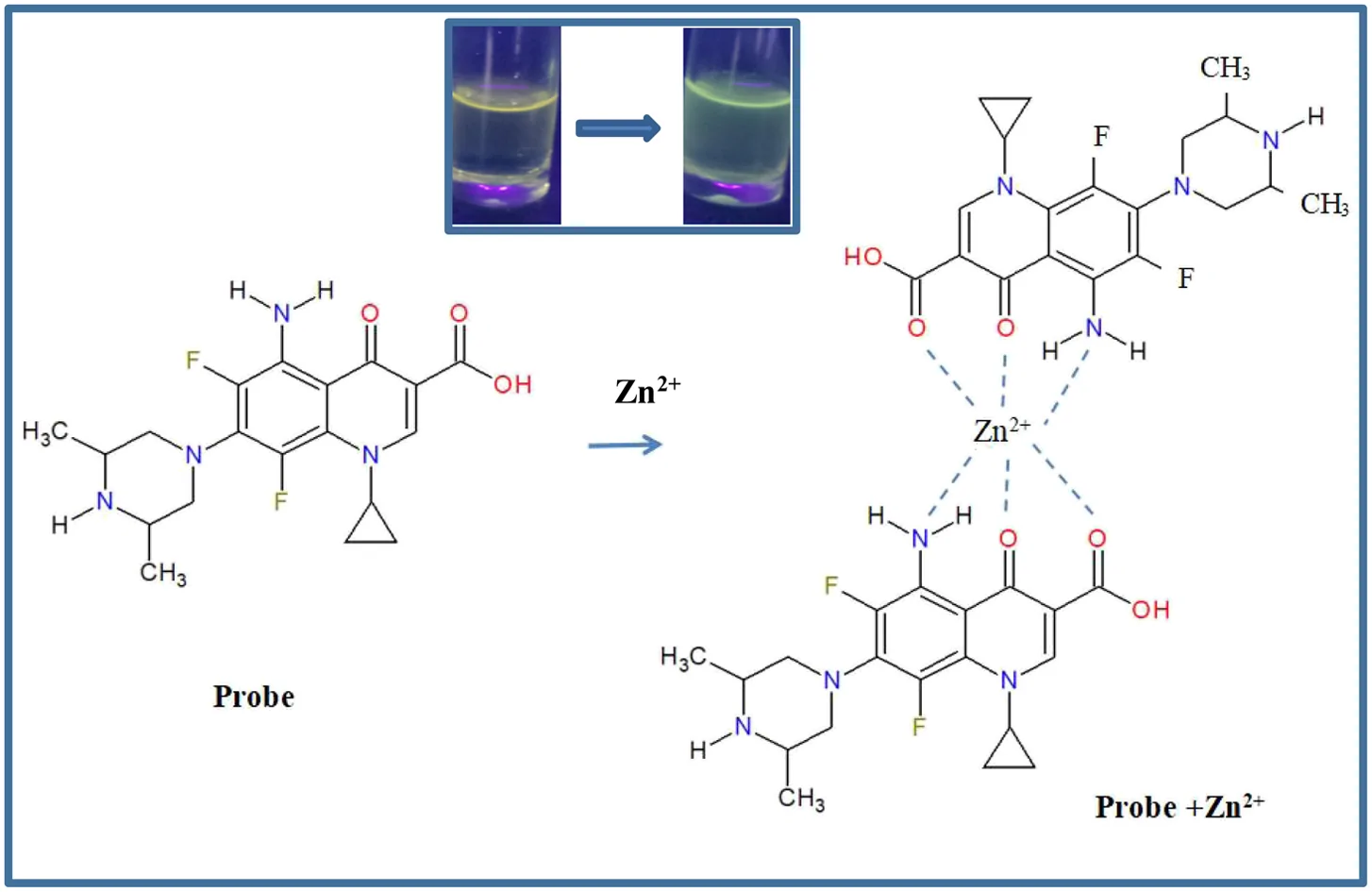

## 1 Introduction

Environmental pollution has emerged as a significant global concern due to its detrimental effects on ecosystems and human health. A wide range of pollutants, including heavy metals, pesticides, insecticides, herbicides, fungicides, synthetic dyes, *p*-nitrophenol, hydrazine, and other hazardous chemicals, are continuously released into the environment, posing serious risks to agricultural productivity and ecological balance. The detection and quantification of these contaminants are commonly performed using electrochemical and optical sensing techniques owing to their high sensitivity, selectivity, and rapid response.^1–3^ Among these pollutants, heavy metals are of particular concern because they are persistent, non-biodegradable, and frequently accumulate in groundwater and other water resources, especially in areas influenced by industrial discharge and anthropogenic activities. In recent years, considerable research attention has been directed toward heavy metal ions because of their significant environmental and biological implications.^4–6^ Metal ions are indispensable for numerous physiological processes, and essential trace elements are required to maintain normal cellular functions and metabolic homeostasis. However, excessive exposure to toxic heavy metal ions can lead to severe environmental contamination and adverse health effects, even at very low concentrations. Consequently, the development of reliable and sensitive analytical methods for the detection and monitoring of metal ions has become an important area of research. Zinc ions are fundamental components in living organisms, performing important roles such as functioning as structural cofactors in metalloproteins, modulating enzyme activity, cellular metabolism, gene transcription and enzyme regulation,^7–9^ and aiding in the transmission of nerve signals. Additionally zinc metal plays multiple roles in several biological processes, namely oxygen transport, energy production and cellular metabolism. Uncontrolled levels of zinc affect the human body easily and lead to some major issues such as prostate cancer, Friedreich's ataxia, and neurodegenerative diseases including Alzheimer's disease, Parkinson's disease and epilepsy.^10–13^ While zinc is indispensable for maintaining good health, consuming it in large quantities may result in zinc poisoning. Toxic effects generally arise when zinc intake surpasses 225 mg. Additionally, an overabundance of zinc can interfere with the body's ability to absorb copper and iron.

The existence of free zinc ions in aqueous environments presents a considerable toxicity threat to a wide range of organisms, including bacteria, plants, invertebrates, and vertebrate fish. According to the Food and Agriculture Organization (FAO) and the World Health Organization (WHO), the acceptable zinc (Zn) content in drinking water is 3 mg L^−1^. Exceeding this level can cause the water to become opalescent and form a greasy film when boiled. The recommended zinc concentration in irrigation water is 2 mg L^−1^ as Zn^2+^. Higher concentrations can be toxic to plants and may render the water a contaminant.^14,15^ High concentrations of free Zn^2+^ ions can cause numerous health problems in mammals, impacting the cardiovascular system, development, immune response, liver and kidney function, as well as neurological, blood-related, pancreatic, and reproductive systems. Therefore, it is crucial to create sensors that can accurately detect Zn^2+^ for applications in environmental surveillance and clinical diagnostics.

Zinc is the second most abundant transition metal in the human body. It plays a fundamental role in numerous physiological and biochemical processes, including gene expression, enzyme activation, apoptosis, immune function, and neuronal signal transduction. The dysregulation of Zn^2+^ homeostasis has been associated with several pathological conditions, including the formation of amyloid-β plaques implicated in Alzheimer's disease. According to the recommended dietary guidelines in the United States, the daily zinc intake is 8 mg for adult women and 11 mg for adult men. Despite its biological importance, the accurate assessment of zinc deficiency remains a significant challenge, particularly in children from developing regions of Africa and Asia, owing to the limited availability of highly selective and sensitive biochemical probes for Zn^2+^ detection.^16^ Consequently, substantial research efforts have been devoted to the development of molecular probes capable of the rapid, selective, and sensitive detection of Zn^2+^ ions.

In polluted environments, metals and metalloids frequently exist together in multiple inorganic and organic forms, ranging from simple hydrated species to complex large organic compounds. Zinc is a vital trace element essential for metabolic processes, serving as a constituent of numerous enzymes, hormones, and factors involved in nucleic acid transcription. Both pediatric and adult research has consistently found abnormally low levels of zinc in patients facing critical illnesses. Additionally, traditional detection techniques such as electronic absorption spectroscopy, electron paramagnetic resonance (EPR), and nuclear magnetic resonance (NMR) fail to identify Zn^2+^ ions, as these ions do not produce signals in these methods. To measure heavy metal concentrations, several sophisticated techniques have been developed, including inductively coupled plasma-mass spectrometry (ICP-MS),^17^ ion-selective electrodes (ISE),^18^ and atomic absorption spectroscopy (AAS) and electrochemical stripping method.^19,20^ However, these approaches require costly equipment and skilled personnel to operate effectively. Moreover, these methods often involve considerable delays due to the need to transport samples to centralized laboratories, which limits their practicality and sometimes makes them unsuitable. In recent times, fluorescent chemical sensors have gained prominence as effective devices for metal ion detection, providing benefits such as exceptional sensitivity, high selectivity, and ease of use. They allow for real-time monitoring and precise identification of specific metal ions. Sensors developed for detecting Zn^2+^ usually incorporate fluorophores and their derivatives, such as coumarin, anthracene, and quinolone. Fluorescence-based techniques have attracted considerable attention from researchers because of their notable advantages, including immediate detection, straightforward visualization, user-friendly operation, excellent selectivity, and remarkable sensitivity.

Herein, amino-1-cyclopropyl-7 (*cis*-3,5 dimethyl-1-piperazinyl)-6,8-difluoro-1,4-dihydro-4-oxo-3-quinoline carboxylic acid (ADQC) (Fig. 1a) is a fluoroquinolone antibiotic commonly prescribed to treat a range of bacterial infections. Probe (ADQC) is a synthetic fluoroquinolone broad-spectrum of antimicrobial agent it has the *in vitro* activity against a wide range of Gram-negative and Gram-positive microorganisms. This probe exerts its antibacterial activity by inhibiting DNA gyrase, a bacterial topoisomerase. DNA gyrase is an essential enzyme which controls DNA topology and assists in DNA replication, repair, deactivation, and transcription.

**Fig. 1:**
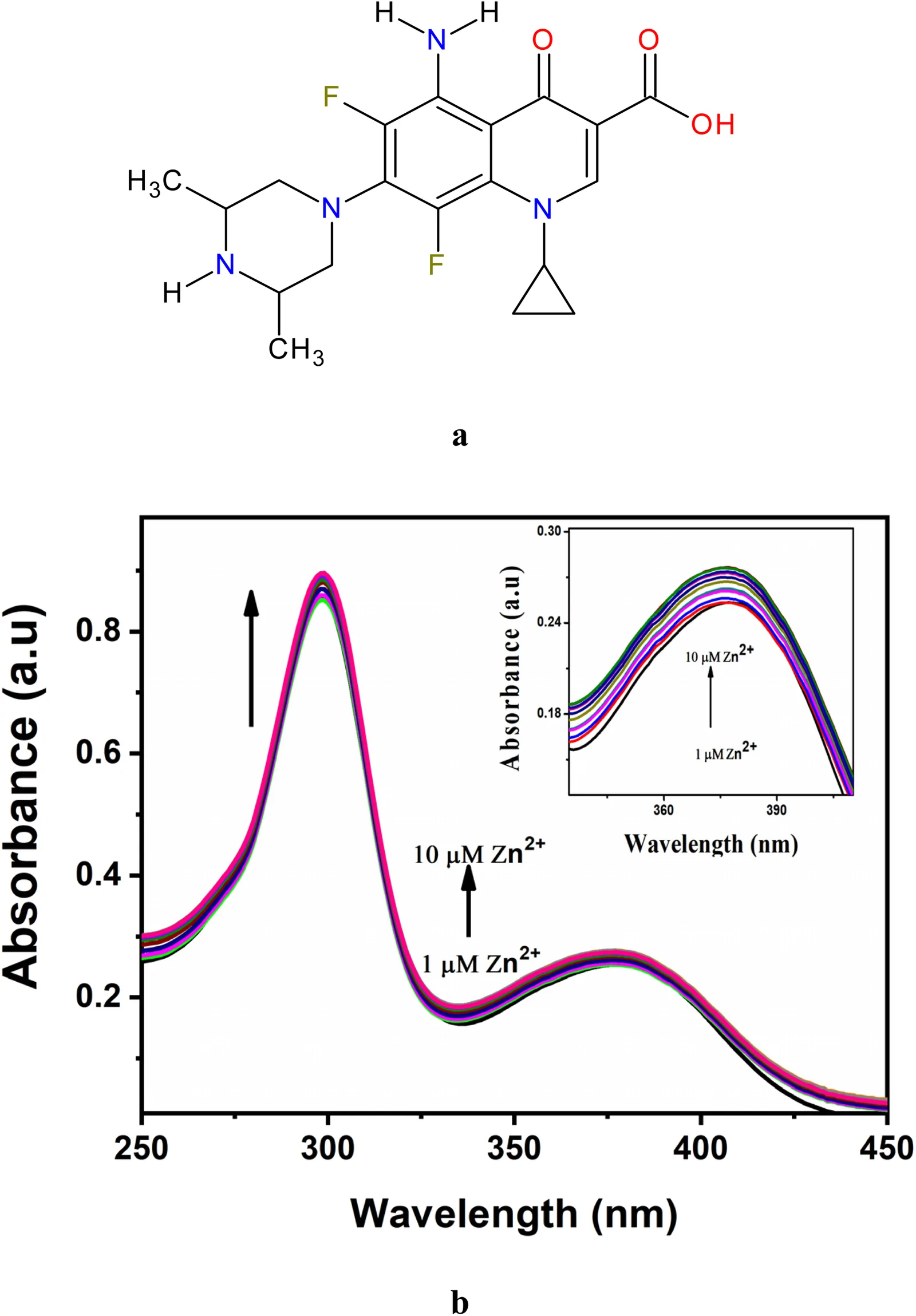
(a) Chemical structure of amino-1-cyclopropyl-7 (*cis*-3, 5 dimethyl-1-piperazinyl)-6, 8 difluoro-1, 4-dihydro-4-oxo-3-quinoline carboxylic acid (ADQC); (b) UV-visible spectra of the probe with increasing Zn^2+^ concentrations (1–10 µM). Inset: expanded region showing the probe–Zn^2+^ absorption maximum at 379 nm.

The above ADQC chemical employed as a novel chemosensor to detect Zn^2+^ in this study. We confirmed that Zn^2+^ ions addition only enhances the fluorescence of fluoroquinolone, remaining ions have no such enhancement was observed, indicating a good selectivity. The sensor achieved a remarkably low detection limit of 1 nM for Zn^2+^ ion compared with earlier reported sensors. In this fluoroquinolone-based probe, Zn^2+^ ions readily coordinate with the carbonyl oxygen and heterocyclic nitrogen atoms. The coordination process leads to the formation of a stable five- or six-membered metallacyclic chelate ring through the involvement of two oxygen atoms and one nitrogen atom. The strong affinity of Zn^2+^ toward oxygen- and nitrogen-donor ligands facilitates the formation of this coordination complex. The practical applicability of the chemosensor was further investigated through live-cell fluorescence imaging experiments using A549 cells.

Zinc (Zn) is an essential micronutrient, but excessive levels can cause cytotoxicity and environmental harm. Free zinc ions (Zn^2+^) in water are toxic to bacteria, plants, invertebrates, and fish, disrupting calcium balance and inflaming gills. Human activities have increased zinc levels in soils, leading to plant accumulation and toxicity. Zinc salts mainly affect the environment through the release of Zn^2+^ ions. Toxicity is influenced by water hardness, pH, and dissolved organic carbon, with the highest risks in soft, neutral, low-DOC waters. Accurate detection and monitoring of zinc ions are vital for environmental protection.

## 2 Experimental

### 2.1 Materials and methods

All reagents used in the experiments were of analytical grade. Solvents such as acetonitrile, acetone, dimethyl sulfoxide, tetrahydrofuran, ethyl acetate, dimethylformamide, and dichloromethane were procured from Merck. Metal acetate salts were also sourced from Merck. The compound amino-1-cyclopropyl-7-(*cis*-3,5-dimethyl-1-piperazinyl)-6,8-difluoro-1,4-dihydro-4-oxo-3-quinoline carboxylic acid was purchased from Sigma-Aldrich and used without further purification. Ultrapure water was obtained from a Millipore system. Additionally, ethanol was purified using standard procedures before use. UV/Vis absorbance measurements were performed using an Agilent UV/Vis spectrophotometer. pH values were measured with a EUTECH pH Tutor, equipped with a glass pH electrode. Fluorescence spectra were recorded using a Cary Eclipse spectrofluorometer, featuring a xenon lamp as the excitation source. Fluorescence lifetime measurements were performed using a time-resolved spectrofluorometer (HORIBA FluoroLog-3). All experiments were conducted at least three times (*n* = 3), and the results are presented as mean ± standard deviation (SD). All the figures were drawn using OriginPro software. As the present study focuses on the analytical performance of the fluorescence probe and does not involve any formal mean separation test (*e.g.*, ANOVA) was performed.

### 2.2 Optical test and data analysis

The UV-vis absorption spectra of the probe (30 µM) in ethanol were recorded using an Agilent 89090A spectrophotometer with a 1 cm path length quartz cuvette at room temperature, both in the absence and presence of Zn^2+^ ions. The absorbance values were measured over the wavelength range of 250–500 nm. Similarly, the fluorescence spectra of the probe in ethanol were recorded using a Cary Eclipse spectrofluorometer, with and without the addition of metal ions. The fluorescence intensity of the probe–metal mixtures was measured over the wavelength range of 400–750 nm under an excitation wavelength of 379 nm. The responses to potential interfering ions were also carefully examined. The stoichiometric binding ratio between Zn^2+^ and the probe was determined based on fluorescence intensity using Job's plot analysis.

In the pH experiments, the pH of the solutions was maintained with appropriate buffer systems to ensure stable, controlled conditions. In particular, buffer solutions were prepared at the time of analysis to minimize fluctuations in pH during the tests and to measure the fluorescence response at different pH values. For real water sample analysis, the collected samples (wastewater and drinking water) were initially filtered to obtain clear solutions. The filtered water samples were then mixed with the probe solution in an ethanol–water mixture (70 : 30, v/v). The pH of the resulting solution was adjusted to and maintained at 6.0 using dilute 0.1 M HCl or 0.1 M NaOH prior to fluorescence measurements. Under these conditions, the calibration curve was plotted for the determination of Zn^2+^ ions in the real sample analysis. The usability of the proposed probe in real water samples was measured by using standard addition method. The known concentration of Zn^2+^ were spiked into the prepared real sample solution, the related fluorescence intensities were recorded and determined the Zn^2+^ ions concentration from the calibration curve. Finally the recovery percentage was calculated for accuracy of the sensing system. The same prepared real solution were also analysis using AAS instrument.

### 2.3 Cell imaging studies

To examine the applicability of the proposed sensor for detecting Zn^2+^ ions in biological systems, fluorescence imaging study was performed. The human lung adenocarcinoma cell line (A549) was procured from the National Centre for Cell Science (NCCS), Pune, India, through an authorized local supplier and purchased by the School of Biotechnology, Madurai Kamaraj University, Madurai, India. The cell line was maintained and handled by trained research scholars following standard cell culture procedures and was subsequently used for cell imaging experiments. A549 cells were seeded in 12 well plates in complete culture media and incubated for 12 h in a CO_2_ incubator. The cells were then treated with the probe and further incubated for 12 h before fluorescence imaging analysis. Upon reaching 80% of its confluency, cells were treated with probe: Zn^2+^ solution (optimized fluorescence concentration) at assigned IC_50_ concentration for 1 h. After that, the treated cells slowly washed three times with phosphate buffer solution (PBS) to remove excess Zn^2+^ ions and were visualized using a Confocal fluorescence microscope equipped with a excitation filter.

## 3 Result and discussion

### 3.1 Absorption studies

The chemical sensor shows specific optical properties in its prepared state. The UV-visible spectra for the probe in an ethanol solution are given in Fig. 1b, which possesses different absorption peaks in the ranges of 260–320 nm and 325–400 nm. The first maximum absorption peak appeared at 298 nm, corresponding to the π–π* transition^21^ of the conjugated carbon–carbon units (C=C) in the aromatic ring and the C=O bond. The another peak center appeared at 380 nm represents the functional groups of the probe indicating the nonbonding to antibonding orbital transition, *i.e. n–*π* transitions of the carbonyl or amine functional groups existing on the probe.^22^ The Zn^2+^ ions was detected by the probe in the UV-vis spectra with the gradual addition of Zn^2+^ ions concentration, as shown in (Fig. 1). The corresponding absorbance peak at 298 and 379 nm peaks were gradually enhances because of the fluoroquinolone complex formation with Zinc. UV-vis spectra was continuously recorded which the addition of Zn^2+^ ion from 1 µM–10 µM with probe at room temperature.

### 3.2 Effect of solvent

The effect of different solvents on the emission spectra of the probe was recorded in the range of 400–750 nm. In this study, we investigated the intermolecular interactions between the probe and various solvents. Two types of solvents were selected based on their hydrogen bond donor (HBD) index: the first group with *α* = 0 (non-HBD solvents) and the second group with *α* ≠ 0 (HBD solvents). The results show that the interaction between the probe and the second group of solvents are significantly influenced by the hydrogen bond donor ability of the solvents, which had a greater effect on the emission behavior than other polarity parameters.^23^ Ethanol exhibits hydrogen bond donor behavior, which enhances the emission response of the probe compared to polar aprotic solvents like DCM and THF. As a polar protic solvent, ethanol can readily donate labile hydrogen atoms, facilitating stronger interactions with the probe. Generally, such solvents possess high dielectric constants and polarity. Therefore, ethanol was chosen as the solvent for dissolving the probe during testing, not only due to its effectiveness but also because it is an environmentally friendly (green) solvent. Fig. 2 clearly illustrates the effect of solvent polarity on the emission spectra. As generally expected, an increase in solvent polarity leads to a bathochromic (red) shift in the fluorescence spectra. This observation confirms that the electronic transition involved is of π–π* character, and that the probe's emission is more pronounced than its absorption. The energy distribution in the ground state remains largely unaffected due to the relatively low polarity of the probe in its ground state, whereas the excited state exhibits a stronger charge transfer character, making it more sensitive to solvent polarity.^24,25^

**Fig. 2:**
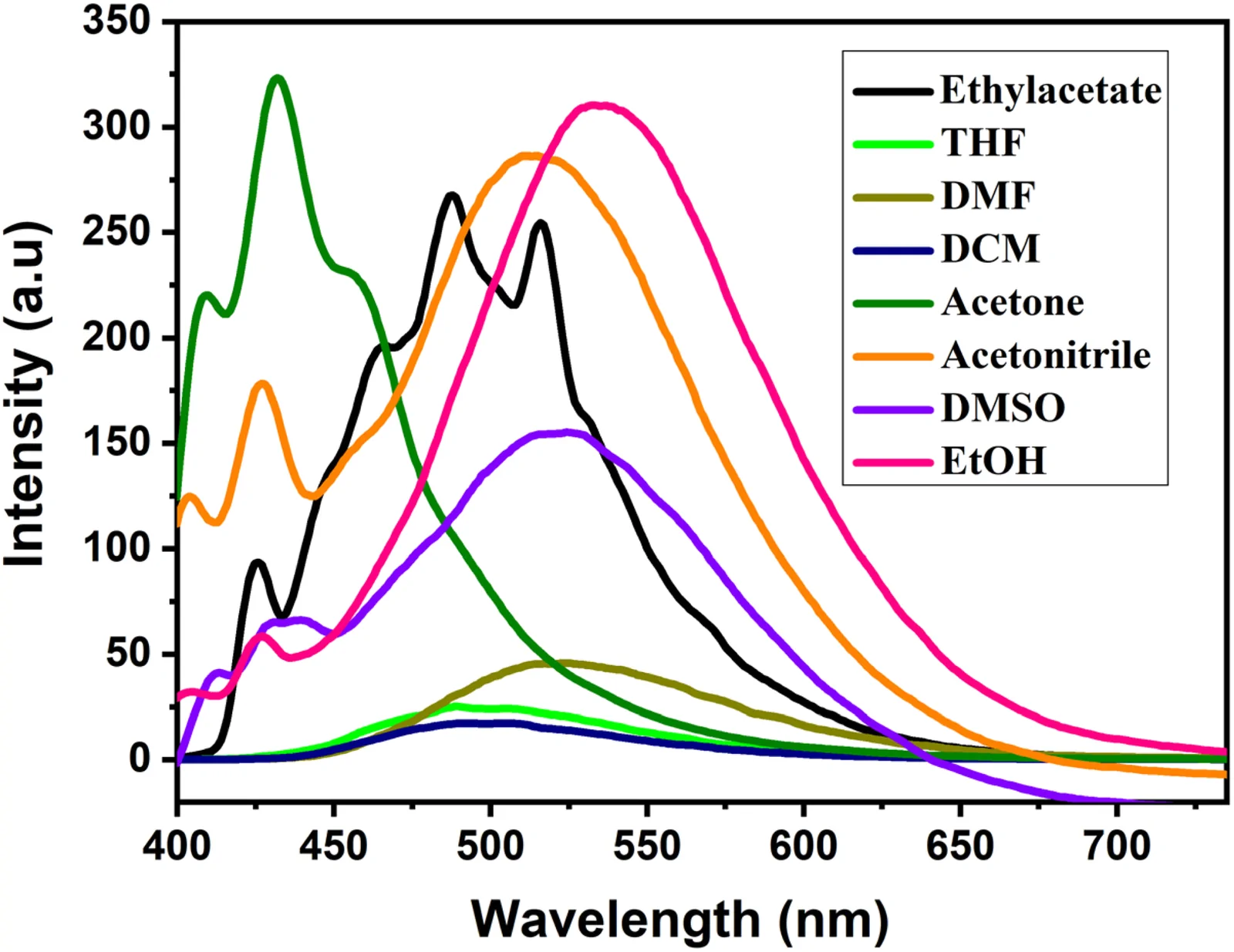
Fluorescence emission spectra of the probe in the presence of various solvents.

### 3.3 Effect of pH

Fig. 3 shows the emission spectral changes of the probe from 400 to 700 nm monitored in media of varying pH. The fluorescence intensity of the probe gradually increases as the pH changes from 1 to 4, which is attributed to the protonation of the electron-donating amine group. At low pH (around 1), the probe exists in a fully protonated form. As the pH increases from 1 to 4, partial deprotonation occurs, leading to an increase in fluorescence intensity due to stabilization of the excited state. However, when the pH is raised further from 5 to 11, complete deprotonation may take place, potentially resulting in aggregation or formation of non-fluorescent species, which leads to a decrease in the probe's fluorescence intensity. At high pH, the electron-donating ability of the amine functional groups in the probe is reduced, leading to a weaker intramolecular charge transfer in the excited state. This increases the energy gap between the ground and excited states, resulting in a blue shift in the emission spectrum.

**Fig. 3:**
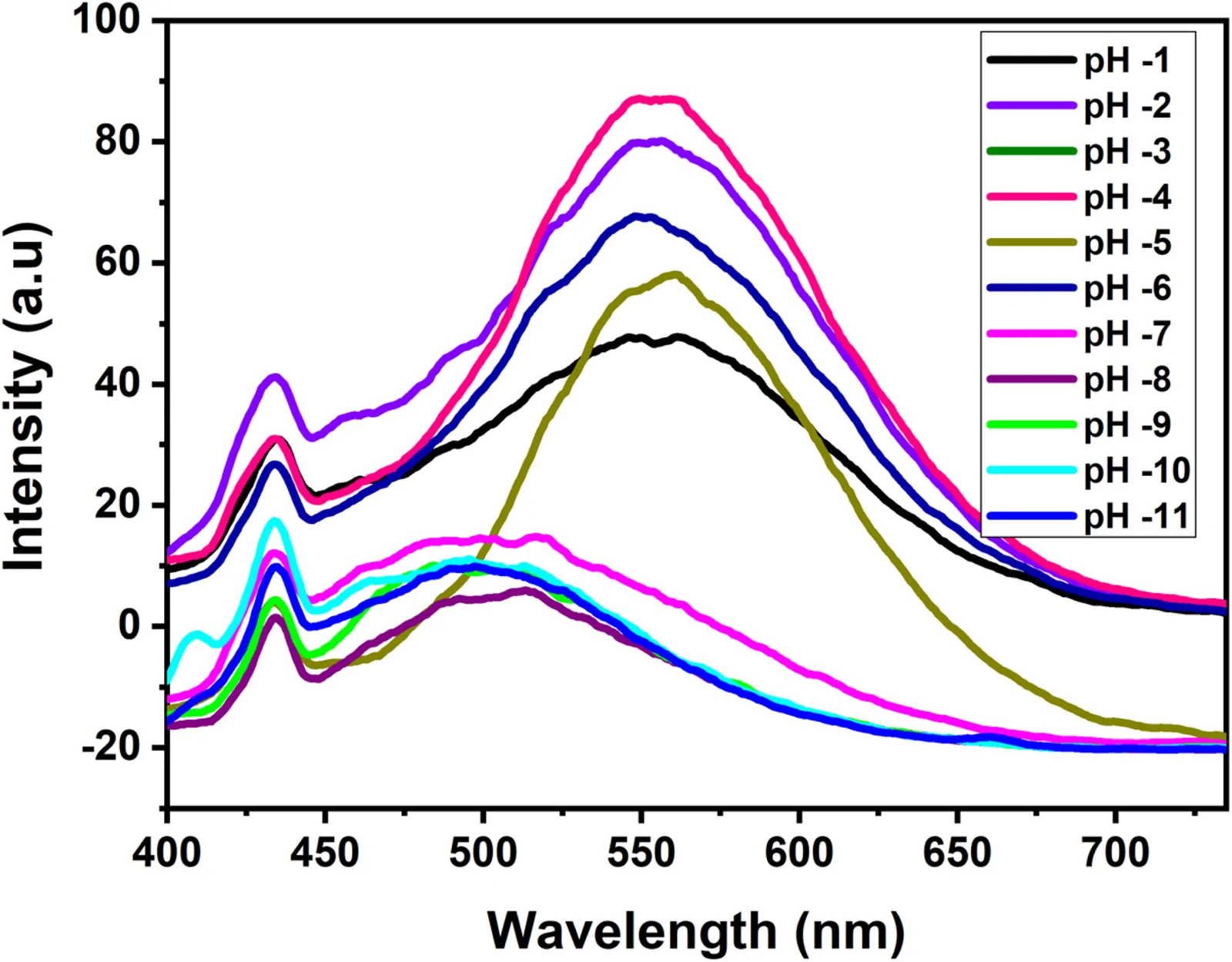
Fluorescence emission spectra of probe recorded at various pH solution.

### 3.4 Selectivity

The selectivity of the probe toward various metal ions, including Na^+^, Ag^+^, Ba^2+^, Ca^2+^, Cd^2+^, Cr^3+^, Hg^2+^, K^+^, Pb^2+^, and Zn^2+^, was studied in an ethanol solution, as shown in Fig. 4. The probe exhibited low fluorescence emission at 538 nm in the absence of metal ions. However, fluorescence titration with Zn^2+^ revealed a significant enhancement in emission intensity, accompanied by a blue shift in the emission maximum from 538 nm to 518 nm (a shift of 20 nm). This response is attributed to the strong interaction of Zn^2+^ with the amine and carboxyl functional groups present in the probe. However, no significant changes in fluorescence intensity were observed upon the addition of other metal ions, indicating the high selectivity of the probe toward Zn^2+^. This selectivity is further supported by a visible color change from light yellow to fluorescent green upon binding with Zn^2+^ (inset: Fig. 4). A slight quenching of fluorescence was observed with the addition of Ag^+^, Ca^2+^, Ba^2+^, and Hg^2+^ ions, whereas only a slight fluorescence enhancement was noted in presence of Cd^2+^, this behaviour can be attributed to the fact that cadmium (Cd^2+^) and zinc (Zn^2+^) belong to the same group of the periodic table and therefore exhibit similar chemical and electronic properties. Both ions possess fully filled d^10^ electronic configurations and comparable ionic radii, enabling them to interact with electron-rich donor atoms. As a result, fluorescence probes often exhibit responses toward both Zn^2+^ and Cd^2+^, making their selective discrimination a significant challenge in environmental and biological monitoring. Conventional approaches typically employ masking agents to suppress the response of one ion and facilitate the selective detection of the other, particularly for monitoring toxic Cd^2+^ in the presence of Zn^2+^. In the present study, interference experiments revealed only a negligible fluorescence response toward Cd^2+^, whereas Zn^2+^ produced a significant enhancement in fluorescence intensity. These results demonstrate that the proposed fluorescence probe exhibits high selectivity toward Zn^2+^ over Cd^2+^ without the need for masking agents. Notably, the probe functions act as a “Turn-on” sensor for Zn^2+^ and is capable of effectively distinguishing Zn^2+^ from Cd^2+^—a differentiation that has traditionally been difficult to achieve.^26^

**Fig. 4:**
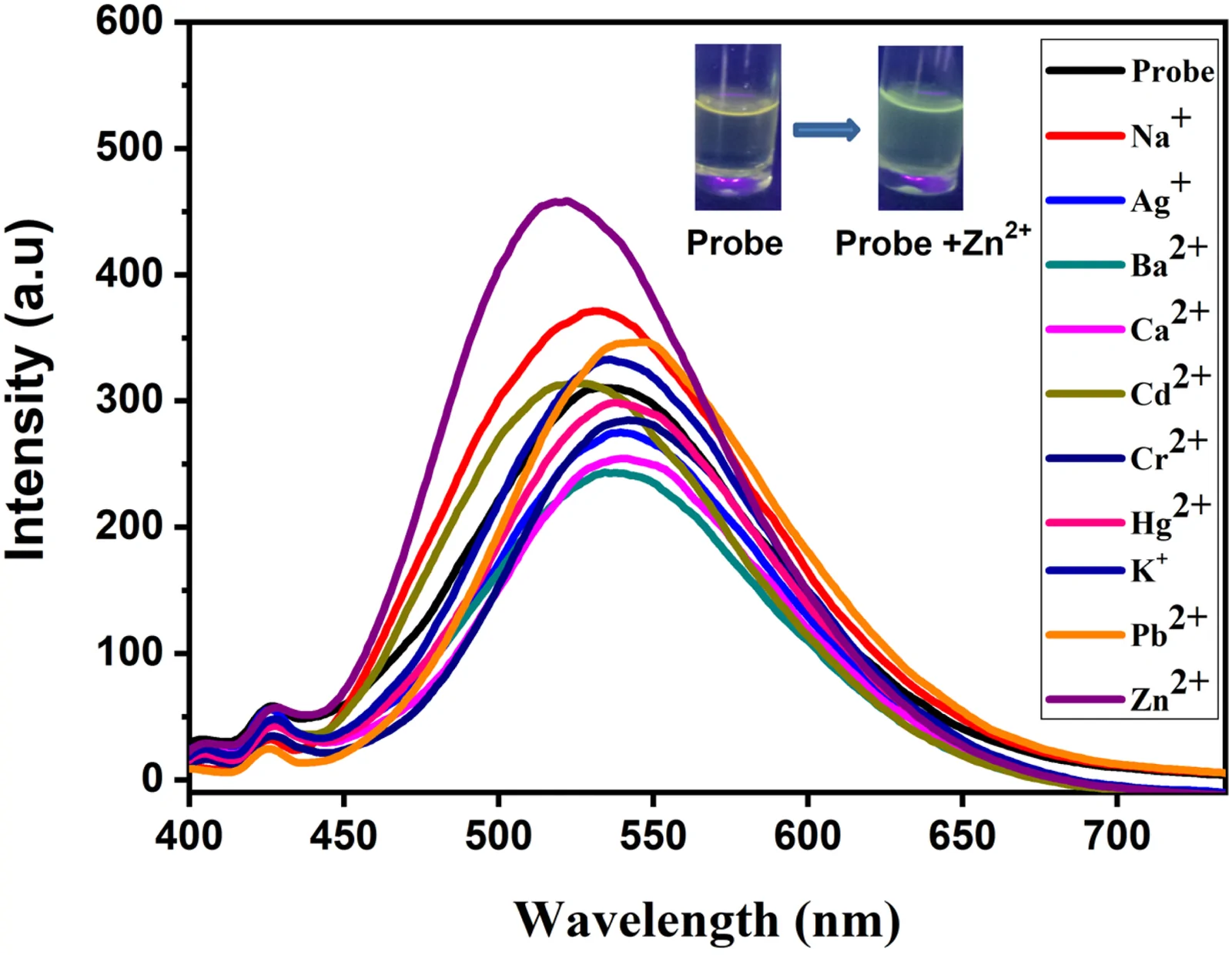
Fluorescence spectra of the probe (30 µM) in the presence of Zn^2+^ ions and other metal ions (12 µM each). Inset: the image shows the color change of the probe and the probe–Zn^2+^ complex as observed by the naked eye.

To further investigate the practical relevance of the probe as a selective chemosensor for Zn^2+^, a competition experiment was conducted. In this experiment, the probe was tested in the presence of Zn^2+^ ions (2 µM) mixed with various other metal ions (Na^+^, K^+^, Ag^+^, Cd^2+^, Pb^2+^, Co^2+^, Ba^2+^, Cu^2+^, Fe^2+^, Ni^2+^, and Cr^3+^) each at a concentration of 4 µM, as shown in Fig. 5. Interestingly, the results show that there were no significant changes in the fluorescence intensity of the Zn^2+^ response, even in the presence of highly concentrated interfering metal ions. This indicates that the probe can be used as a highly selective sensor for Zn^2+^ detection. Moreover, it can effectively differentiate between Zn^2+^ and other metal ions, particularly distinguishing Zn^2+^ from Cd^2+^ ions, which exhibit many similar behaviors.

**Fig. 5:**
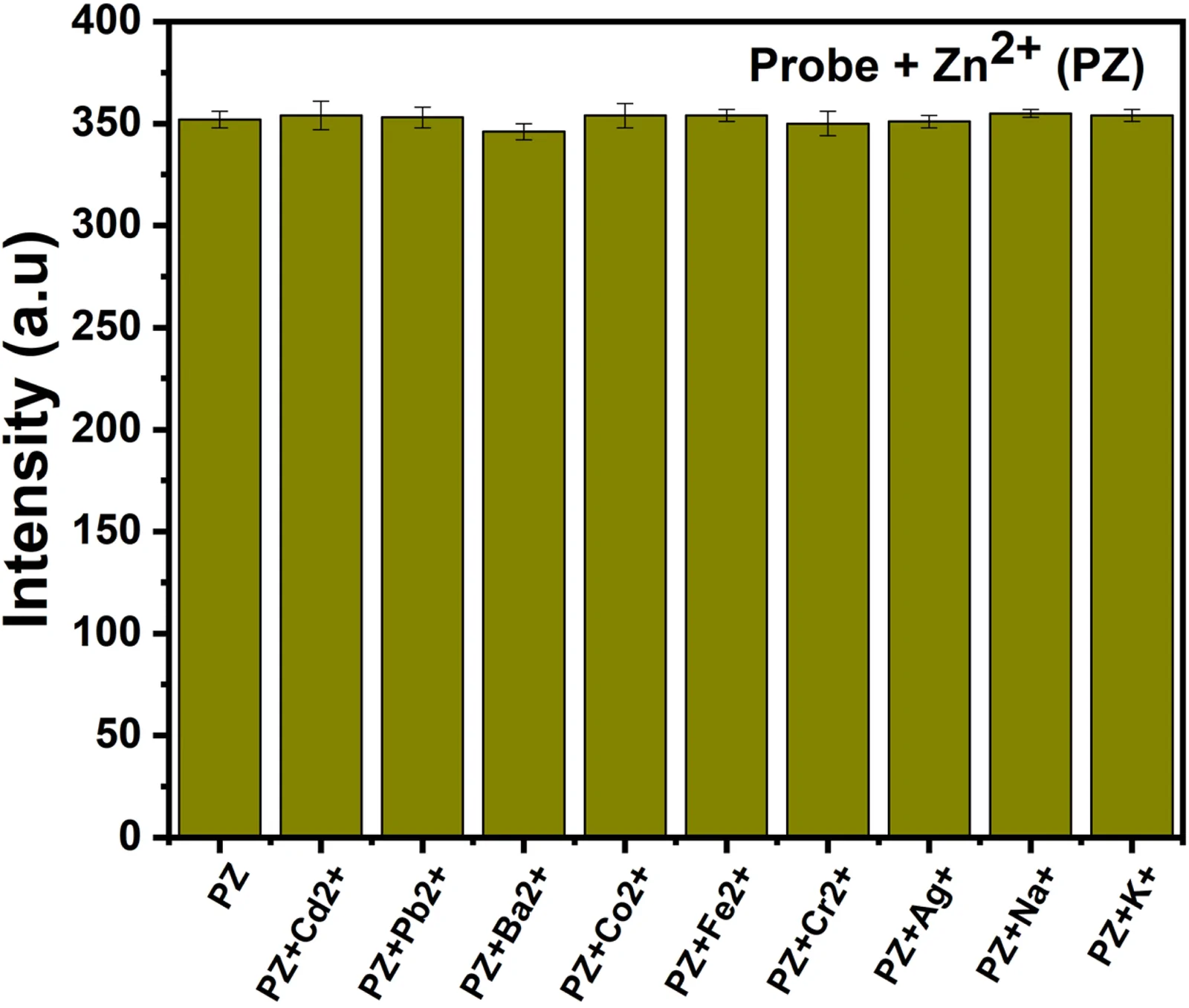
Bar diagram of fluorescence intensity of the probe (30 µM) in the presence of Zn^2+^ ions (2 µM) and with other interfering metal ions (4 µM each).

### 3.5 Sensitivity and optical properties

The effectiveness of the fluorescent sensor probe was evaluated through the quantitative analysis of Zn^2+^ ions. As shown in Fig. 6a–c, when the concentration of Zn^2+^ ions was gradually increased from 1 nM to 15 µM while keeping the probe concentration constant at 30 µM, the fluorescence intensity increased continuously. This clearly demonstrates the probe's sensitivity toward Zn^2+^ ions. At higher concentrations, the fluorescence intensity approached a saturation level. Under optimal conditions, a good linear relationship was observed between Zn^2+^ concentration (1 nM–15 µM) and fluorescence intensity. The fluorescence intensity of the blank sample was measured, and the standard deviation was calculated. The calibration plot for Zn^2+^ detection is shown in Fig. 6b. Based on the equation 3*σ*/*k* (where *σ* is the standard deviation of the blank and *k* is the slope of the calibration curve),^27,28^ the detection limit for Zn^2+^ was calculated to be 1 nM. This value is lower than those reported in previous studies (as summarized in Table 1) and is also below the acceptable limit for drinking water set by the U.S. EPA.^43^ The data in the table confirm that the proposed probe is more sensitive than previously reported sensors. The chemosensor demonstrates sensitivity to Zn^2+^ concentrations as low as 1 nM. Therefore, the developed probe is suitable for the selective and sensitive detection of Zn^2+^ ions.

**Fig. 6:**
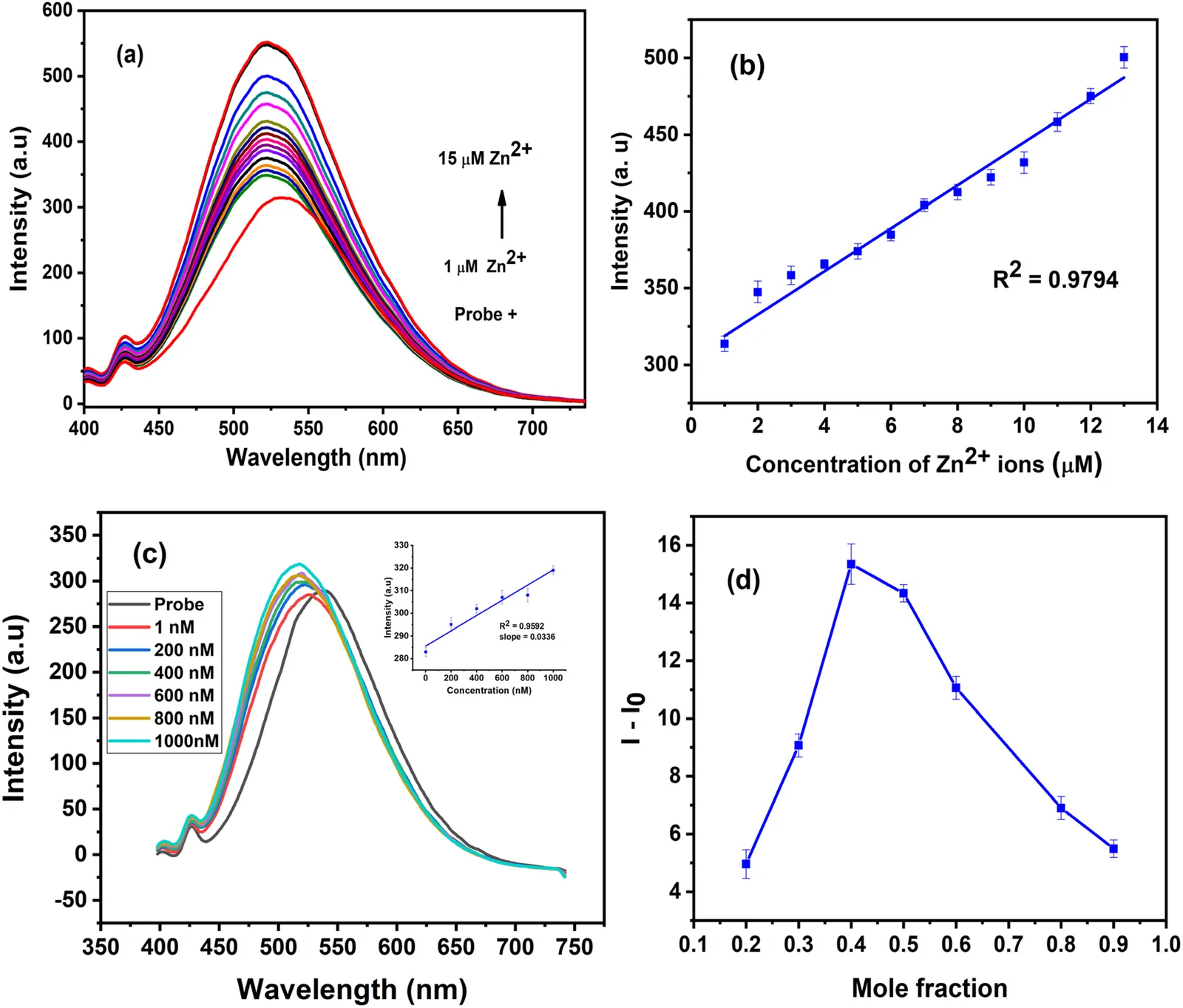
(a) Fluorescence spectra of the Zn^2+^–probe complex obtained at different Zn^2+^ ion concentrations ranging from 1 µM to 15 µM, (b) the corresponding calibration plot of fluorescence intensity *versus* Zn^2+^ concentration, and (c) fluorescence spectra of the Zn^2+^–probe complex recorded at Zn^2+^ ion concentrations ranging from 1 nM to 1000 nM, (d) Jobs plot for complex formed between probe and Zn^2+^ ions.

**Table 1:** Comparison data with other reported fluorescence probes for zinc ion detection with turn-on signal

Response	Solvent	Metal (Zn^2+^) (LOD)	Excitation (nm)	Emission (nm)	Mechanism	Reference
Turn-on	Tris–HCl buffer (CH_3_OH/H_2_O = 9 : 1, v/v)	0.144 µM	430	516	PET and CHEF effect	29
Turn-on	Ethanol/HEPES buffer (95/5, v/v)	0.503 µM	242	450	(i) Restriction in rotation of the CQN group,	30
					(ii) blockage of the PET process,	
					(iii) Zn^2+^ induced disaggregation of sensor 50	

Turn on	DMF	8.6 nM	430	509	CHEF effect and CQN isomerization	31
Turn on	DMF–H_2_O (9 : 1, v/v)	2.5 µM	380	500	PET process and CQN isomerization	32
Turn on	Ethanol	1.75 µM	430	498	PET process, CQN isomerization and CHEF effect	33
Turn on	Ethanol	0.134 µM	510	572	Opening of the lactam ring	34
Turn on	DMSO–H_2_O (1 : 9, v/v, HEPES buffered)	0.52 µM	450	525	ICT	35
Turn on	Éthanol–H_2_O buffered (9 : 1, v/v)	1.2 nM	330	512	PET process, CQN isomerization and CHEF effect	36
Turn on	HEPES buffered ethanol–H_2_O (9 : 1, v/v)	1.59 µM	375	520	PET process, CQN isomerization and CHEF effect	37
Turn on	DMSO/HEPES (v/v, 1 : 1) buffer	0.69 µM	391	458	Inhibition of ESIPT process	38
Turn on	Acetonitrile and HEPES buffer (1 : 1, v/v, pH 7.4)	1 nM	—	—	PET blocking	39
Turn on	Aqueous solution-pH 7.2	70 nM	401	470	—	40
Turn on	Ethanol	0.75 µM	378	450	Formation of coordination complex	41
Turn on	CH_3_CN: H_2_O	5 nM	454	563	Excited state 320 intramolecular proton transfer (ESIPT) is responsible for the quenching of fluorescence	42
**Turn on**	Ethanol	**1 nM**	379	518	ICT	**This study**

The sensing mechanism is attributed to the binding of Zn^2+^ ions to the electron-donating site, specifically the lone pair of electrons on the nitrogen atom of the amine group. The fluorophore contains an electron-donating group conjugated to an aromatic benzene ring, which facilitates intramolecular charge transfer (ICT) from the donor to the acceptor upon light excitation.^44,45^ When the electron-rich terminal of the probe, particularly the oxygen atom of the carboxyl group interacts with Zn^2+^ ions, this interaction influences the absorption or excitation wavelength of the fluorophore by altering its ICT excited state.^46^ This interaction likely reduces electron donation, alters the intramolecular electron flow, and enhances intramolecular charge transfer (ICT), resulting in increased fluorescence intensity along with a blue shift in the emission wavelength.

The stoichiometry of the probe–Zn^2+^ complex was determined using Job's plot, based on varying mole fractions (Fig. 6d). The maximum emission intensity was observed at a mole fraction of 0.4, indicating the formation of a 2 : 1 ligand-to-metal complex, as illustrated in Scheme 1. Devasia, *et al.* reported a similar 2 : 1 binding stoichiometry for a 2-(4,5-diphenyl-1*H*-imidazole-2-yl)pheno-based probe–Cu^2+^ complex.^47^ Based on the Job's plot, the binding constant (*K*) of the probe with Zn^2+^ was calculated using the Benesi–Hildebrand equation,^43,48^ yielding a value of 3.39 × 10^1^ M^−1^. This binding constant falls within the typical range reported for Zn^2+^-binding chemosensors that exhibit nonlinear relationships,^49^ and is consistent with the general range of 0.3 to 7.09 observed for Zn^2+^ chemosensors. The detection limit of Zn^2+^ is far below the WHO guidelines for drinking water.^50,51^

**Scheme 1:**
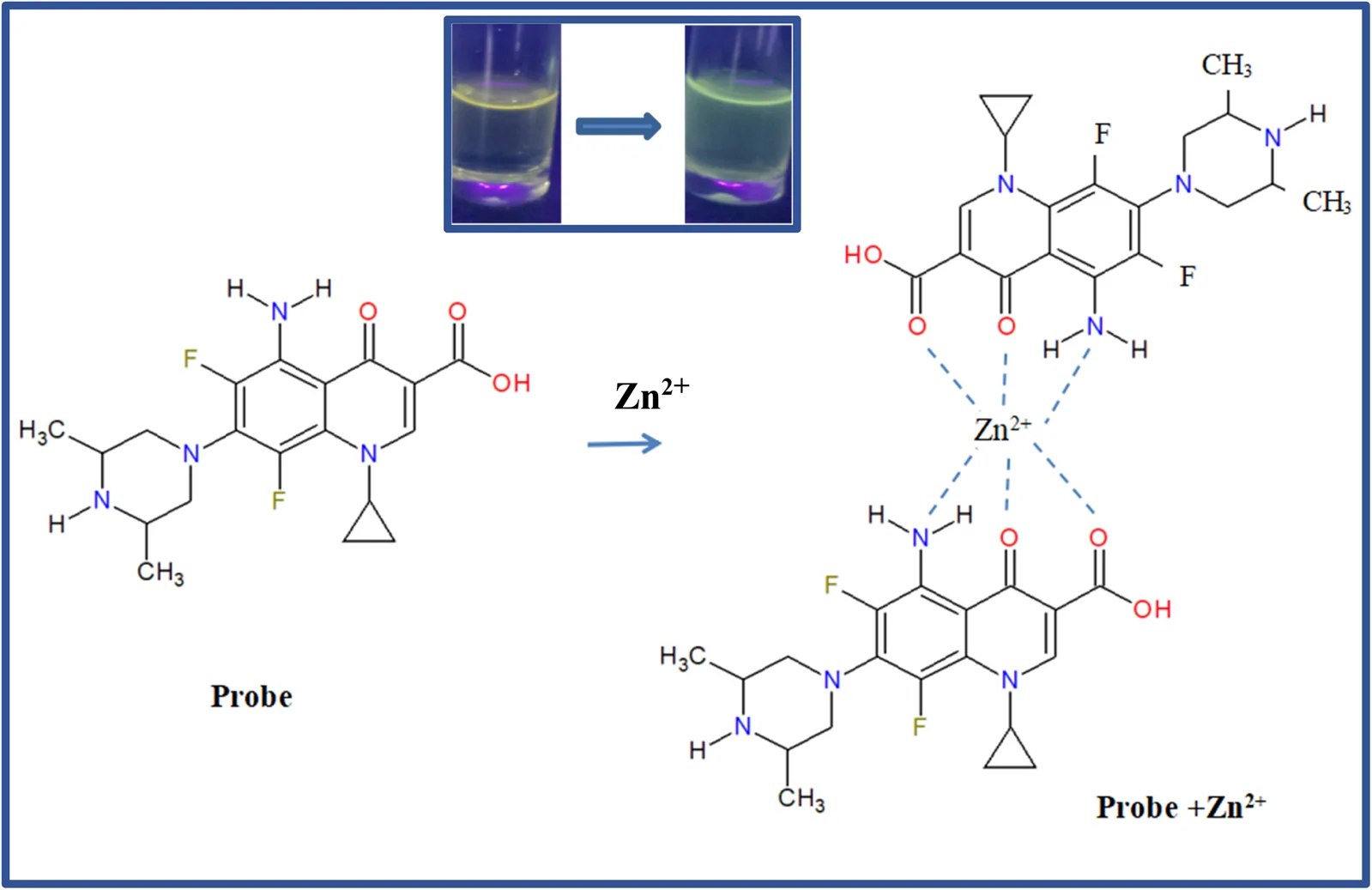
Schematic representation of the complexation of the probe with Zn^2+^ ions.

The relative standard method is one of the common methods to identify the fluorescence quantum yield (*Φ*) of unknown fluorophore by compare the value with known standard compound. This method is very simple and accurate method in fluorescence studies. Here the standard fluorophore Rhodamine 6G (quantum yield: 0.95) in ethanol solution was used for this study as a reference due to the excitation wavelength is close to the probe. In this method, the absorbance of the probe and standard solution at excitation wavelength was regulated below 0.1 for reduce the inner filter effects. The measurement of the fluorescence test must be same instrument with identical conditions, then the quantum yield of probe can be measured by using the following relative quantum yield (*Φ*_*x*_) equation.
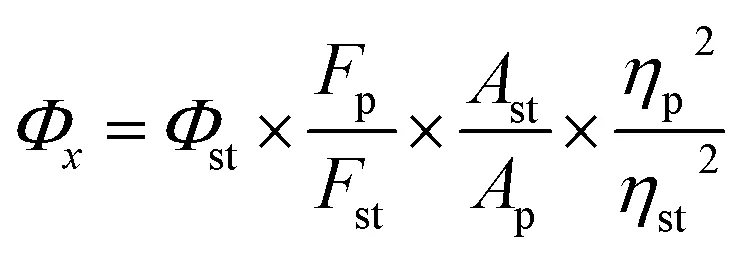
where *A* is the absorbance, *F* is the integrated fluorescence intensity of the standard and probe, and *η* is the solvent's refractive index. The subscripts (St) and (p) denote to the standard Rhodamine 6G and probe, respectively, and since both measurements were performed under the same solvent conditions, *η*_p_/*η*_st_ is nearly equal to the 1.0. The proposed probe exhibited a fluorescence yield (*Φ*_*x*_) of ∼0.45 which is less than the Rhodamine 6G standard (*Φ* ∼0.95) under the same experimental condition.

Time-resolved fluorescence spectroscopy was employed to investigate the excited-state dynamics of the free probe and its Zn^2+^ complex (Fig. 7a). The fluorescence decay profiles of both the free probe and the Zn^2+^-bound probe were best fitted with a biexponential decay model. The average fluorescence lifetime increased from 1.80 ns for the free probe to 3.34 ns upon complexation with Zn^2+^, indicating that Zn^2+^ coordination effectively suppresses non-radiative decay pathways and stabilizes the excited state. Fluorescence lifetime spectroscopy (time-resolved fluorescence) is a significant method to support an intramolecular charge transfer (ICT) sensing mechanism. The observed increase in fluorescence lifetime upon Zn^2+^ coordination indicates the suppression of non-radiative ICT relaxation pathways and stabilization of the ICT excited state, resulting in enhanced fluorescence emission. These results provide strong evidence for an ICT-assisted sensing mechanism in the proposed Zn^2+^ fluorescent probe. The ICT mechanism is also supported by solvatochromic studies (Fig. 2), as evidenced by changes in emission wavelength with solvent polarity (a hallmark of push–pull chromophores). The red shift with increasing order of solvent polarity indicating the stablization of the excited ICT state in polar medium. The compounds with strong ICT show large solvatochromic shifts, making them suitable for polarity probes and environment-sensitive fluorophores.

**Fig. 7:**
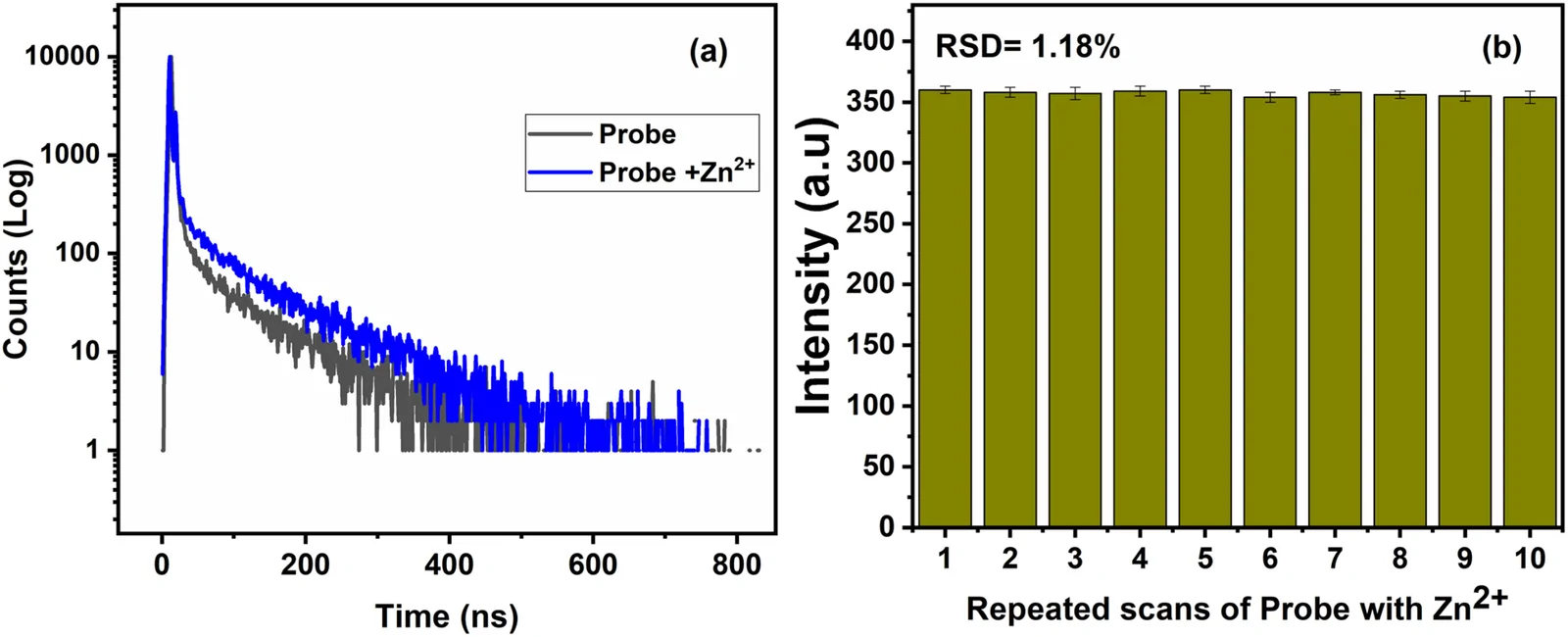
(a). Time-resolved fluorescence decay of probe, and probe-Zn^2+^complex; (b) repeatability of the fluorescence sensor for the detection of Zn^2+^ ions over 10 replicates.

We evaluated the repeatability of the proposed sensor for the detection of Zn^2+^ ions in ethanol. Fig. 7b shows the repeatability measurements (10 consecutive tests) of Zn^2+^ detection using the fluorescence method, which exhibited a low relative standard deviation (RSD) of 1.18%. These results demonstrate that the developed sensor can be reliably used for repeatable detection of Zn^2+^ ions.

The fluorescence responses of the probe were investigated in ethanol–water mixtures of varying compositions (Fig. 8), including pure ethanol (100%), ethanol : water (70 : 30, v/v), and ethanol : water (30 : 70, v/v). The results clearly indicate that the ethanol–water composition significantly influences the fluorescence emission of the probe, owing to continuous changes in solvent polarity, probe solubility, and hydrogen-bonding interactions. In the ethanol : water 70 : 30 v/v mixture, the probe exhibited enhanced solubility and more effective coordination with Zn^2+^ ions, resulting in a stronger fluorescence response compared to that observed in the ethanol : water (30 : 70, v/v) system. In contrast, the higher water content in the 30 : 70 mixture increases the polarity of the medium, which reduces the fluorescence response of the probe and promotes molecular aggregation and hydrogen-bonding interactions, leading to fluorescence quenching. At low water content (ethanol : water, 70 : 30 v/v), a pronounced red shift was observed, indicating favorable solvation of the probe in the ethanol-rich medium. In contrast, at high water content (ethanol : water, 30 : 70 v/v), only a slight red shift (lower than expected) along with peak broadening was observed. This behavior can be attributed to the high polarity of water, which reduces the fluorescence intensity of the probe and may promote intermolecular interactions, such as aggregation and hydrogen bonding.

**Fig. 8:**
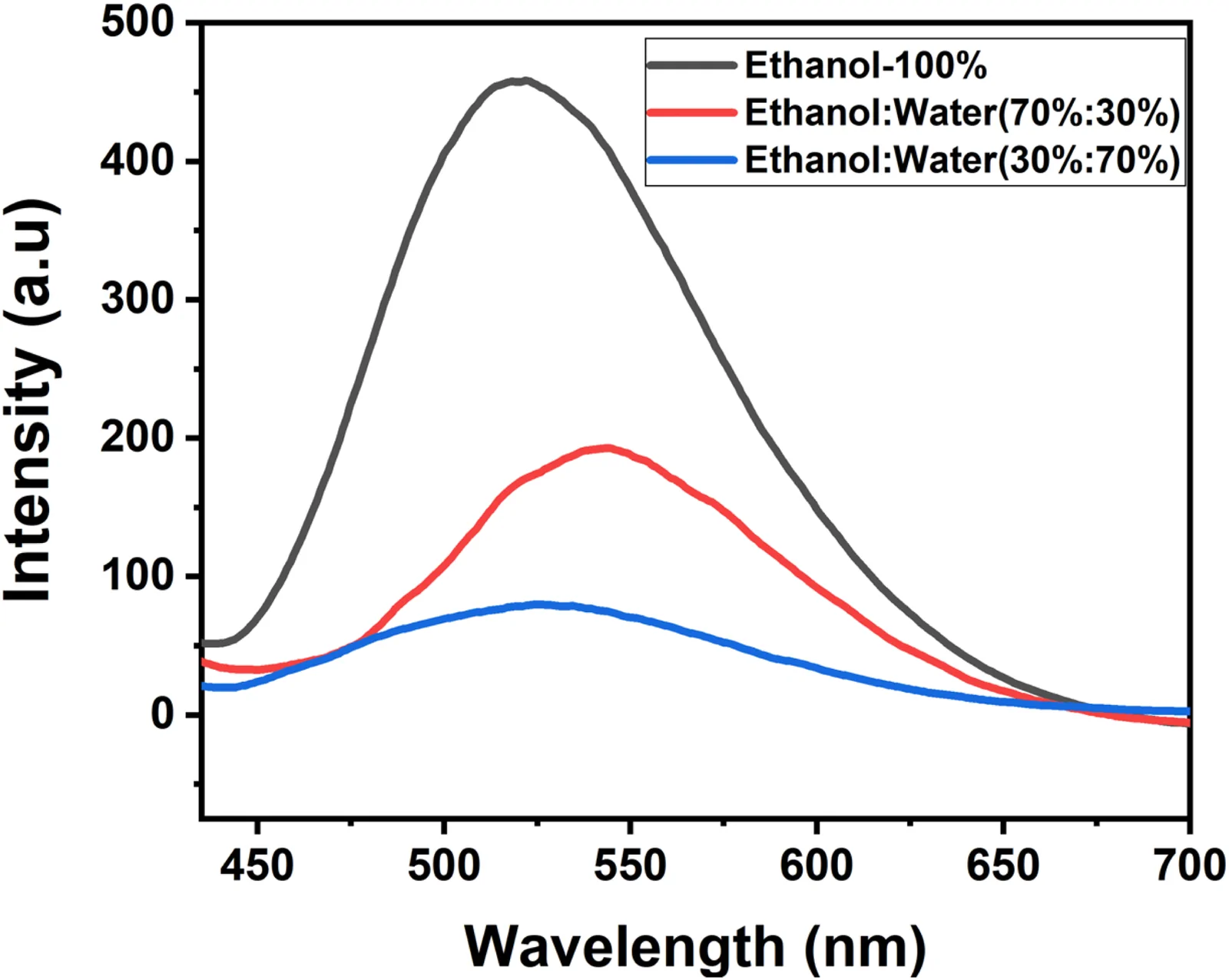
Effect of the ethanol–water composition on fluorescence sensor.

The fluorescence cell imaging studies demonstrated the biocompatibility of the proposed probe and indicated its promising potential for the detection of Zn^2+^ ions in living cells. The fluorescence images shown in Fig. 9 confirm the non-toxic nature of the probe toward cultured A549 cells. Cells treated with the probe alone exhibited only weak fluorescence, indicating a low background signal in the absence of Zn^2+^ ions. However, A549 cells treated with probe in the presence of Zn^2+^ions shows significant enhancement in fluorescence, as observed from the images captured using the Confocal fluorescence microscope. These results further confirmed that proposed sensor is cell-permeable and can be used for monitor the Zn^2+^ ions in living cells.

**Fig. 9:**
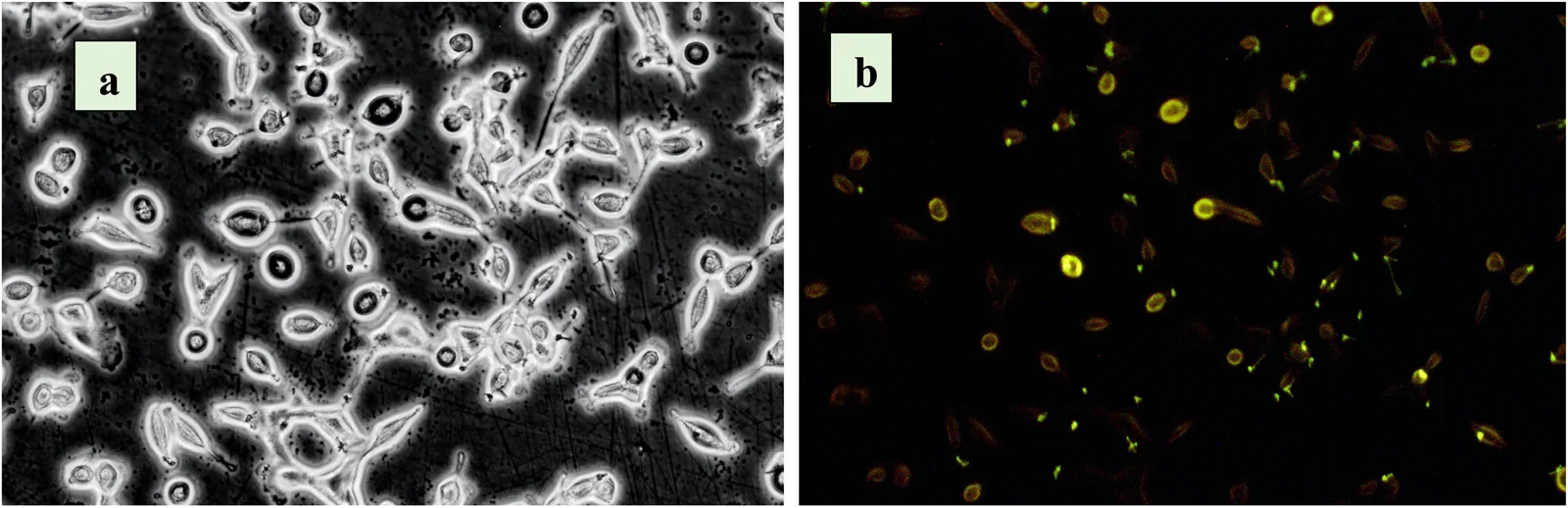
Confocal fluorescent image studies of A549 cells alone (a) and probe: Zn^2+^ ions (b) under optimized concentration.

The Zn^2+^ ion detection ability of the chemosensor was evaluated in real environmental water samples using the spiked metal detection method. The recovery percentage was determined by fluorescence emission measurements in both drinking water and waste water samples, calculated using a linear regression equation, and presented in Table 2. All experiments were repeated in three times, and the results showed good recovery rates ranging from 97.0% to 102.75%. This results finally compared with AAS results. These findings demonstrate that the sensor is suitable for practical Zn^2+^ detection in real samples, even in the presence of other competing metal ions.

**Table 2:** Results for zinc ions determination in real water samples

Samples	Initially found (µM)	Spiked Zn^2+^ ions (µM)	Found Zn^2+^ ions (µM) (±SD, *n* = 3)	Recovery (%) using this method	RSD (%) (*n* = 3)	Recovery (%) using AAS
Waste water	0.09	1	1.12 ± 0.03	102.7%	2.85%	101.9%
	0.10	2	2.05 ± 0.06	97.6%	3.01%	98.0%
	0.12	5	5.18 ± 0.2	101.2%	3.87%	100.6%
Drinking water	0	1	0.97 ± 0.02	97.0%	1.50%	99.5%
	0	2	1.97 ± 0.05	98.5%	2.35%	99.8%
	0	5	4.98 ± 0.15	99.6%	2.97%	99.5%

## 4 Conclusion

In summary, we developed an optical sensor specifically designed for the detection of zinc ions (Zn^2+^) with a turn-on signal, focusing mainly on its sensitivity, selectivity, and response time. This sensor offers several advantages for heavy metal detection in real samples, including an easy preparation method, low-cost reagents, minimal instrumentation requirements, and scalability. Notably, the sensor enables rapid and naked-eye detection within seconds or minutes, depending on the Zn^2+^ concentration. We successfully detected Zn^2+^ ions using UV-visible and fluorescence spectroscopy, achieving a fast response time of 3 minutes. The sensor exhibited a broad linear range from 1 nM to 15 µM with a detection limit of 1 nM. Optical studies revealed that the proposed sensor demonstrates superior performance for both individual and simultaneous detection of Zn^2+^ ions, exhibiting excellent selectivity and high sensitivity—capable of detecting Zn^2+^ concentrations as low as 1 nM. Moreover, it can effectively differentiate between Zn^2+^ and other metal ions, particularly distinguishing Zn^2+^ from Cd^2+^ ions, which exhibit many similar behaviors. The sensing mechanism based on the interaction between probe and Zn^2+^ likely reduces electron donation, alters the intramolecular electron flow, and enhances intramolecular charge transfer (ICT), resulting in increased fluorescence intensity along with a blue shift in the emission wavelength. The sensor offers excellent reproducibility and superior selectivity, even in the presence of interfering ions at twice the concentration of the target Zn^2+^ ions. Further investigation of the sensor's performance in various solvents and pH conditions yielded acceptable results. We successfully validated the sensor using real water samples, demonstrating its practical applicability. Therefore, the proposed sensor can be effectively used for detecting Zn^2+^ ions in polluted and industrial wastewater, as well as in biomedical applications. In the present study, a few limitations should be acknowledged. The fluorescence studies were carried out using an ethanol-based solvent system to prepare the probe solution. Although this solvent provided good solubility and sensing performance, the development of a fully aqueous-based fluorescent probe for Zn^2+^ detection would significantly broaden its practical applicability, particularly for biological and environmental samples. Further studies are currently in progress to expand the environmental and real-world sensing applications of this probe for Zn^2+^ detection. Future work will focus on the highly sensitive determination of Zn^2+^ in complex food matrices for food safety monitoring, as well as the detection of Zn^2+^ in biological samples and biomolecules under physiologically relevant conditions.

## Author contributions

Daniel Arulraj Abraham: writing – review & editing, writing – original draft, validation, supervision, resources, project administration, methodology, investigation, funding acquisition, formal analysis, conceptualization; Ravikumar Ayyanu: effect of different ethanol–water ratios on evaluating the probe and fluorescence quantum yield studies; Ellairaja Sundaram: fluorescence lifetime measurement and fluorescence analysis, data analysis and interpretation; G. Balasubramani: cell imaging studies, data analysis, and interpretation of the study; A. Anand Babu Christus: real sample analysis, data analysis and interpretation of the results.

## Conflicts of interest

The authors declare no conflict of interest.

## Data Availability

Data will be made available on request

## References

[cit1] Raj A. D. A., Vijayan M., Vasantha V. S. (2015). Highly selective and sensitive simple sensor based on electrochemically treated nano polypyrrole-sodium dodecyl sulphate film for the detection of para-Nitrophenol. Anal. Chim. Acta.

[cit2] Raj A. D. A., Vijayan M., Vasantha V. S. (2015). Spectrophotometric Determination of Pico-Molar Level of Hydrazine by using Alizarin Red in Water and Biological Sample, Spectro. Acta Part A: Mole. Biomolec. Spe..

[cit3] Raj A. D. A., Vasantha V. S. (2020). Development of electrochemically treated conducting polymer- based sensor for the detection of fenuron. ACS Omega.

[cit4] Li H. Q., Li Y., Liu Y. T., Deng S. W., Wang W., Li S. Y., Wang Z. Y. (2026). Fluorescent Detection Probes for Hg2+ and Zn2+ with Schiff Base Structure Based on a Turn-On ESIPT–CHEF Mechanism. Chemosensors.

[cit5] Kaur M., Virender V., Singh J., Kumar A., Dubey S. K. (2025). Smartphone-based analytical platform for real-time, reversible detection of Co^2+^, Ni^2+^, Cu^2+^, and Zn^2+^ ions. Food Chem..

[cit6] VinothKumar G. G., Bhaskar R., Harathi J., Fernando R., Borda C., Hussain S. S., Sundara moorthy A. (2023). A simple fluorescent sensor for the meticulous recognition of Cu2+ ion and its functioning in logic gate and keypad lock. J. Photochem. Photobiol. A: Chem..

[cit7] Kim H. J., H Lee M. (2026). A Dual-functionalized naphthalimide fluorescent probe for selective zinc detection and imaging in live prostate cancer cell. Dyes Pigm..

[cit8] Yu J., Jiang T., Lin Z., Yu H., Wang S., Qi Y. (2025). Two novel pyrene-based Schiff base fluorescent probes for the turn-on detection of Zn2+ and their application in bioimaging. Microchem. J..

[cit9] Chasapis V. C., Loutsidou A. C., Spiliopoulou C. A., Stefanidou M. (2012). Zinc and human health: an update. Arch. Toxicol..

[cit10] Sperotto R. A., Boff T., Duarte G. L., Santos L. S., Grusak M. A., Fett J. P. (2010). Identification of putative target genes to manipulate Fe and Zn concentrations in rice grains. J. Plant Physiol..

[cit11] Yang H., Peng Y., Haung L., Zhang H., Wang Y., Xie S. (2013). Synthesis and characterization of CaMoO_4_:Eu^3+^ red phosphors. J. Lumin..

[cit12] Ashraf M., Firmansyah M. L., Azeim S. A., Ullah N. (2026). The design of β-carboline–8-aminoquinoline tethered probes for nanomolar Zn^2+^ sensing and cell imaging. Sens. Actuators, B.

[cit13] Chen B., Yu P., Nok Chan W., Xie F., Zhang Y., Liang L., Leung K. T., Lo K. W., Yu J., Tse G. M. K., Kang W., Fai K. (2024). Cellular zinc metabolism and zinc signaling: from biological functions to diseases and therapeutic targets. Signal Transduction Targeted Ther..

[cit14] Noulas C., Tziouvalekas M., Karyotis T. (2018). Zinc in soils, water and food crops. J. Trace Elem. Med. Biol..

[cit15] Xia Y., Zhou W., Zhong Y., Zhao Z., Wen J., Ding S. (2025). A theoretical study on the ICT mechanism of zinc ion fluorescent probe based on triphenylamine. Spectrochim. Acta, Part A.

[cit16] Kare A. P., Assefa T., Desalegn B. B., Astatkie A. (2025). Prevalence and factors associated with zinc deficiency among preschool-age children in rural districts of Sidama region, Ethiopia: A community-based cross-sectional study. PLOS Glob. Public Health.

[cit17] Verma P., Kalra N., Verma S. (2024). Advancement in sensory identification of heavy metal contamination in water: A review on progression from spectroscopic analytical techniques to handheld sensors. Microchem. J..

[cit18] Wang S.-L., Sukesan R., Hsieh C.-Y., Wang Y.-L. (2018). Heavy Metal Ion Detection from Whole Blood Using Ion Selective FET Sensor. ECS Trans..

[cit19] Raj A. D. A., Rajkumar D., Chen S.-M., Vasantha V. S. (2016). Femtomolar detection of mercuric ions using Polypyrrole, Pectin and Graphene nanocomposites Modified electrode, Sea-Fue Wang. J. Colloid Interface Sci..

[cit20] Raj A. D. A., Vijayan M., Samseya J., Vasantha V. S. (2014). A simple and highly sensitive electrochemically reduced p-nitrobenzoic acid film-modified sensor for determination of mercury. Electroanalysis.

[cit21] Niu W. J., Li Y., Zhu R. H., Shan D., Fan Y. R., Zhang X. J. (2015). Ethylenediamine-assisted hydrothermal synthesis of nitrogen-doped carbon quantum dots as fluorescent probes for sensitive biosensing and bioimaging. Sens. Actuators B: Chem..

[cit22] Devi S., Kaur A., Sarkar S., Vohra S., Tyagi S. (2018). Synthesis and characterization of highly luminescent N-doped carbon quantum dots for metal ion sensing. Integr. Ferroelectr..

[cit23] Azizi S. N., Chaichi M. J., Yousef M. (2009). Investigation of solvent effects on UV–vis absorption spectra of dihydroxybenzene derivatives by solvatochromic parameters. Spectrochim. Acta, Part A.

[cit24] Tao W., Zhao Z., Kang J., Li Z., Zhu J., Zuo Y., Huang M., Cui J., Yu J., Liu Y., Fang W., Li Y. (2025). Solvent polarity-driven modulation of excited states: A DFT and spectroscopic analysis. J. Photochem. Photobiol. A: Chem..

[cit25] Das D. K., Goswami P., Medhi B. (2013). N-benzoate-N′ salicylaldehyde ethynelediamine: A new fluorescent sensor for Zn^2+^ ion by “off-on” mode. J. Fluoresc..

[cit26] Tan Y., Gao J., Yu J., Wang Z., Cui Y., Yang Y. (2013). Guodong Qian, A new fluorescent probe for distinguishing Zn^2+^ and Cd^2+^ with high sensitivity and selectivity. Dalton Trans..

[cit27] Arulraj A. D., Rajkumar D., Chen S.-M., Vasantha V. S., Wang S.-F. (2015). Highly selective and sensitive fluorescent chemosensor for femtomolar detection of silver ions in aqueous medium. Sens. Bio-Sens. Res..

[cit28] Arulraj A. D., Vijayan M., Vasantha V. S. (2015). Spectrophotometric Determination of Pico-Molar Level of Hydrazine by using Alizarin Red in Water and Biological Sample. Spectrochim. Acta – A: Mol. Biomol. Spectrosc..

[cit29] Dong W., Akogun S. F., Zhang Y., Sun Y., Dong X. (2017). A reversible “turn-on” fluorescent sensor for selective detection of Zn^2+^. Sens. Actuators, B.

[cit30] Zhu J., Zhang Y., Chen Y., Sun T., Tang Y., Huang Y., Yang Q., Ma D., Wang Y., Wang M. (2017). A Schiff base fluorescence probe for highly selective turn-on recognition of Zn^2+^. Tetrahedron Lett..

[cit31] Kumar M., Kumar A., Singh M. K., Sahu S. K., John R. P. (2017). Sens. A novel benzidine based Schiff base “turn-on” fluorescent chemosensor for selective recognition of Zn^2+^. Sens. Actuators, B.

[cit32] Liu C., Zhou Q., Li Y., Garner L. V., Watkins S. P., Carter L. J., Smoot J., Gregg A. C., Daniels A. D., Jervey S., Albaiu D. (2020). Research and Development on Therapeutic Agents and Vaccines for COVID-19 and Related Human Coronavirus Diseases. ACS Cent. Sci..

[cit33] Yan J., Fan L., Qin J., Li C., Yang Z. (2016). novel and resumable Schiff-base fluorescent chemosensor for Zn(II). Tetrahedron Lett..

[cit34] Tang X., Han J., Wang Y., Ni L., Bao X., Wang L. (2017). Zhang, A multifunctional Schiff base as a fluorescence sensor for Fe^3+^ and Zn^2+^ ions, and a colorimetric sensor for Cu^2+^ and applications. Spectrochim. Acta, Part A.

[cit35] Gomathi A., Viswanathamurthi P., Natarajan K. (2019). Fluorescence sensing response of zinc(II) and pyrophosphate ions by benzoxazole appended dipodal Schiff base. J. Photochem. Photobiol., A.

[cit36] Xu Y., Wang H., Zhao J., Yang X., Pei M., Zhang G., Zhang Y., Lin L. (2019). A simple fluorescent schiff base for sequential detection of Zn^2+^ and PPi based on imidazo[2,1-b]thiazole. J. Photochem. Photobiol., A.

[cit37] Das B., Jana A., Das A., Chattopadhyay D., Dhara A., Mabhai S., Dey S. (2019). Fluorescein derived Schiff base as fluorimetric zinc (II) sensor via ‘turn on’ response and its application in live cell imaging. Spectrochim. Acta, Part A.

[cit38] Yuan C., Li S., Wu Y., Lu L., Zhu M. (2017). Zn(II)-selective and sensitive fluorescent chemosensor based on steric constrains and inhibition of ESIPT. Sens. Actuators, B.

[cit39] Kumar M., Kumar A., Singh M. K., Sahu S. K., John R. P. (2017). A novel benzidine based Schiff base “turn-on” fluorescent chemosensor for selective recognition of Zn^2+^. Sens. Actuators, B.

[cit40] Li Y., Li K., He J. (2016). A “turn-on” fluorescent chemosensor for the detection of Zn(II) in aqueous solution at neutral pH and its application in live cells imaging. Talanta.

[cit41] Jin Y., Wang S., Zhang Y., Song B. (2016). Highly selective fluorescent chemosensor based on benzothiazole for detection of Zn^2+^. Sens. Actuators, B.

[cit42] Sarkar D., Pramanik A., Jana S., Karmakar P., Mondal T. K. (2015). Quinoline based reversible fluorescent ‘turn-on’ chemosensor for the selective detection of Zn^2+^: Application in living cell imaging and as INHIBIT logic gate. Sens. Actuators, B.

[cit43] Gupta V. K., Singh A. K., Ganjali M. R., Norouzi P., Faridbod F., Mergu N. (2013). Comparative study of colorimetric sensors based on newly synthesized Schiff bases. Sens. Actuators, B.

[cit44] Melo L., Silva A. M. S., Albuquerque H. M. T. (2025). The role of quinoline in the development of near-infrared fluorescent probes for diagnosis of Alzheimer's disease. Eur. J. Med. Chem..

[cit45] An J., Kim K., Lim H. J., Kim H. Y., Shin J., Park I., Cho I., Kim H. Y., Kim S., McLean C., Choi K. Y., Kim Y., Lee K. H., Kim J. S. (2024). Early onset diagnosis in Alzheimer’s disease patients via amyloid-β oligomers-sensing probe in cerebrospinal fluid. Nat. Commun..

[cit46] Nayak L., Acharya S., Routray S., Pattnaik S., Satapathy R. (2025). Multifunctional s-triazine-BODIPY conjugates: synthetic strategies, photophysical insights, and emerging applications. RSC Adv..

[cit47] Devasia J., Joy F., Nizam A. (2023). A Selective Excited-State Intramolecular-Proton-Transfer (ESIPT) Sensor for Copper(II) Based
on Chelation-Enhanced Quenching and “Off-On” Detection of Amino Acids. Chem. – Eur. J..

[cit48] Roy D., Chakraborty A., Ghosh R. (2017). Perimidine based selective colorimetric and fluorescent turn-off chemosensor of aqueous Cu^2+^: studies on its antioxidant property along with its interaction with calf thymus-DNA. RSC Adv..

[cit49] Wang L., Qin W., Liu W. (2010). Inorg. A sensitive Schiff-base fluorescent indicator for the detection of Zn^2+^. Chem. Commun..

[cit50] Kumar M., Puri A. (2012). A review of permissible limits of drinking water. Indian J. Occup. Environ. Med..

[cit51] Enbanathan S., Munusamy S., Jothi D., Manojkumar S., Manickam S., KulathuIyer S. (2022). Zinc ion detection using a benzothiazole-based highly selective fluorescence “turn-on” chemosensor and its real-time application. RSC Adv..

